# Age and Method of Inoculation Influence the Infection of Worker Honey Bees (*Apis mellifera*) by *Nosema ceranae*

**DOI:** 10.3390/insects10120417

**Published:** 2019-11-22

**Authors:** Almudena Urbieta-Magro, Mariano Higes, Aránzazu Meana, Laura Barrios, Raquel Martín-Hernández

**Affiliations:** 1 IRIAF. Instituto Regional de Investigación y Desarrollo Agroalimentario y Forestal, Laboratorio de Patología Apícola, Centro de Investigación Apícola y Agroambiental (CIAPA), Consejería de Agricultura de la Junta de Comunidades de Castilla-La Mancha, Camino de San Martín s/n, 19180 Marchamalo, Spain; aurbieta@ucm.es (A.U.-M.); mhiges@jccm.es (M.H.); 2 Departamento de Sanidad Animal, Facultad de Veterinaria, Universidad Complutense de Madrid, 28040 Madrid, Spain; ameana@ucm.es; 3 Statistics Department, Computing Center SGAI-CSIC, 28006 Madrid, Spain; 4 Instituto de Recursos Humanos para la Ciencia y la Tecnología (INCRECYT-FEDER), Fundación Parque Científico y Tecnológico de Castilla—La Mancha, 02006 Albacete, Spain

**Keywords:** *Apis mellifera*, *Nosema ceranae*, host-parasite interactions, age of infection, epidemiology, method of infection, parasite load

## Abstract

The microsporidian parasite *Nosema ceranae* is a highly prevalent, global honey bee pathogen. *Apis mellifera* is considered to be a relatively recent host for this microsporidia, which raises questions as to how it affects its host’s physiology, behavior and longevity, both at the individual and colony level. As such, honey bees were inoculated with fresh purified spores of this pathogen, both individually (Group A) or collectively (Group B) and they were studied from 0 to 15 days post-emergence (p.e.) to evaluate the effect of bee age and the method of inoculation at 7 days post-infection. The level of infection was analyzed individually by qPCR by measuring the relative amount of the *N. ceranae* *polar tubule protein 3* (*PTP3)* gene. The results show that the bee’s age and the method of infection directly influence parasite load, and thus, early disease development. Significant differences were found regarding bee age at the time of infection, whereby the youngest bees (new-born and 1 day p.e.) developed the highest parasite load, with this load decreasing dramatically in bees infected at 2 days p.e. before increasing again in bees infected at 3–4 days p.e. The parasite load in bees infected when older than 4 days p.e. diminished as they aged. When the age cohort data was pooled and grouped according to the method of infection, a significantly higher mean concentration and lower variation in *N. ceranae* infection was evident in Group A, indicating greater variation in experimental infection when spores were administered collectively to bees through their food. In summary, these data indicate that both biological and experimental factors should be taken into consideration when comparing data published in the literature.

## 1. Introduction

Beekeeping is a primary sector practice principally aimed at producing nutritive and healthy products for human consumption [1,2], yet it also contributes significantly to the preservation of global biodiversity through the pollinating service that bees provide [2,3]. One of the major threats to *A. mellifera* honey bees are transmissible diseases [4,5]. In this respect, the microsporidian parasite *Nosema ceranae* is a prevalent global parasite of bees [6,7,8,9,10,11] and it is associated with colony weakness and loss, especially in the temperate Mediterranean areas [6,12,13].

This microsporidia is a unicellular obligate parasite that infects the bee’s digestive tract [14]. Infection occurs after ingestion of mature spores in food or water, mainly transmitted by trophallaxis [15] or via the fecal-oral route (fecal waste cleaning) in infected nests [16,17]. Once infection occurs, the parasite proliferates inside the ventricular cells, causing morphological and physiological changes [17,18,19,20], as well as epithelial degeneration [18,19,20]. By exploiting the host cell’s machinery to obtain resources, *N. ceranae* infection affects vital functions and modifies the host’s immune response, ultimately causing cell lysis [21] and digestive disorders, provoking changes in vital functions and modifying the host’s immune response, and significantly compromising the survival of the infected bee [22,23,24,25,26,27,28,29]. This pathogen also affects aspects of honey bee neurobiology, such as olfaction, learning and memory [30], and the orientation and homing skills of workers [31], accelerating bee aging and their related tasks [32,33,34].

Many factors may influence the pathogeny (course and development) of *N. ceranae* infection such as the genetic diversity of honey bees [25,35,36]; beekeeping practices [37,38,39], and climatic and environmental differences [40,41,42,43]. Other factors that could influence the development of the infection are the age of the bees, the means of transmission and the source of the spores. Moreover, for assays performed in a laboratory, the results of the assay may be affected by the specific conditions used, including the duration of the experiment, the temperature, method of infection, humidity, source and type of food, type of cages, number of bees in the cages, wax access, queen hormone, etc. [11,44]. Currently, there is very limited information about the influence of bee age on *N. ceranae* infection. Studies developed under field conditions have demonstrated that older worker bees are the most strongly infected [45,46,47]. By contrast, younger queens were found to be more susceptible than older ones after inoculation with *N. ceranae* spores in the laboratory [48]. However, there is little data regarding the susceptibility of worker bees to infection by this microsporidium. Likewise, it remains unclear how bee age influences the pathophysiology of infection [49], as the age and caste affect the replicative activity of intestinal stem cells in *A. mellifera* [50].

Consequently, we set out to study if the age of worker bees at the moment of exposure to *N. ceranae* spores has any influence on the development of the infection. Similarly, as most studies in apiculture science and insect pathology are performed in laboratory cages under controlled conditions [51] in order to overcome the difficulties in controlling certain variables in open field studies [5,11,34,51,52,53], we evaluated how the method of bee inoculation with *N. ceranae* spores influences the success of infection.

## 2. Materials and Methods

### 2.1. Source of Bees and Rearing Conditions

The experiments presented here were carried out in August–September 2017. To minimize any potential colony-level effects on the results [51], frames of capped brood were obtained from 5 healthy, *Nosema*-free (confirmed by PCR; [54,55]) colonies of *Apis mellifera iberiensis* located in an experimental apiary 20 km from the Centro de Investigación Apícola y Agroambiental (CIAPA) in Marchamalo (Castilla-La Mancha, Marchamalo, Spain). The frames were kept in an incubator at 34 °C (±1 °C) to provide a supply of newly emerged *Nosema*-free honey bees over 15 days. All new-born worker bees were removed daily from the brood combs, they were carefully confined to steel mesh cages [56] in groups of 20 and they were kept in a different incubator (33 °C ± 1 °C) until infection. The age of the bees (days post-emergence: p.e.) was recorded and all the bees in a cage were of the same age (age cohort). The bees were fed ad libitum with a freshly prepared sucrose solution (50% w/w in dH_2_O) supplemented with 2% Promotor L^®^ (Calier Lab., Les Franqueses del Vallès, Spain), a commercial mixture of amino acids and vitamins. Honey and pollen were not used to feed the bees to avoid any possible contamination [57] with infective *Nosema* spp. spores and to avoid problems of standardization. Dead bees were removed daily from the cages and in this way, cages with bee cohorts from 0 to 15 days p.e. were available for *Nosema*-spore inoculation at almost all times.

### 2.2. N. ceranae Spores

Fresh spores were isolated two days before their use from around 200 heavily infected *A. m. iberiensis* bees collected from three naturally infected colonies at CIAPA (see the detailed procedures in Higes et al. [58]). Briefly, groups of 30–40 bees were macerated in dH_2_O (PCR quality) for 120 s at low speed (Stomacher 80-Microbiomaster^®^) in a filter bag (Seward, AK, USA, BA6040). The macerate was centrifuged for 6 min at 800 *g* and the pellet was purified by gradient separation [59] on 95% isotonic Percoll^®^ (in dH_2_O) at a ratio of 1:9 (spores-pellet:Percoll), centrifuging at 11,000 *g* for 40 min. Subsequently, the clean spores were washed three times and recovered by centrifugation at 800 *g* for 6 min, the supernatant was decanted between washes and the sediment was resuspended uniformly in 1 mL sterile ddH_2_O. The species of *Nosema* that the spores corresponded to was confirmed by PCR, as described previously [54,55], and the mean *N. ceranae* spore concentration was obtained by counting the purified spores four times under a light microscope using a Neubauer^®^ haemocytometer as described in [60]. The final spore concentration was established as 57,000 spores µL^−1^ and the stock spore solution was then immediately divided into aliquots (50 µL, spore inoculum), and the vials were stored at room temperature in darkness until they were used two days later.

### 2.3. Infection Experiments: Group A and Group B

Two assays were carried out in parallel. In the first of these, the bees were infected individually (Group A) and in the second, the bees were infected collectively (Group B). Each age cohort (n = 80, 20 bees per cage) from days 0–15 p.e. was assigned to 4 cages and they were distributed in a way that 3 cages were used for the individual infections (Group A) with the fourth cage designated for the collective infection (Group B). In addition, cages with bees of different ages were established (n = 300 bees) for use as a control groups; Group CA and Group CB for Group A and Group B, respectively. These control groups were used to ensure the absence of any initial infection.

Both assays were designed so that infections were only performed on two different days. This strategy allowed the bees of different ages and of the two infection methods to be inoculated on the same day, such that no bias was introduced by the aliquot or through the spore conservation. The bees in the control group were the last ones to be manipulated after inoculating the study groups at both infection times.

All infections took place after bees were starved for 2 h and anesthetized by a 90 sec exposure to CO_2_ for easy manipulation. Regardless of the method of infection, all the bees were inoculated with 114,000 *N. ceranae* spores to promote rapid multiplication such that it could be detected in the breeding conditions employed [25,33,40,47,48,52,61,62,63,64].

#### 2.3.1. Group A: Individual Infection of Bees at Different Ages

Bees from the Group A (3 cages of 20 bees from each age cohort) were held individually by the wings and when they started to wake up, they were administered a 2 µL drop of the spore solution (57,000 spores µL^−1^) through a micropipette [65]. The spore solution was vortexed after every third bee to ensure the suspension remained uniform.

In this assay, bees aged 0 to 15 days p.e. were inoculated (except for those on day 3 that were not available). A control group of bees was included at 0, 1, 4, 5, 8, 11, 13 and 14 days p.e. (1 cage of 20 bees per age), and they were individually fed 2 µL of spore-free water. Bees that did not consume the entire water droplet were discarded and a cardboard barrier was used to physically separate the cages of the different cohorts, avoiding contact between the bees of different ages in the same incubator (Memmert^®^ Mod. IPP500). Infected and uninfected groups of bees were kept in separate incubators under the same conditions (darkness, 33 ± 1 °C and 80% relative humidity). The age cohorts and number of bees used in the study are shown in Table 1.

#### 2.3.2. Group B: Collective Infection of Groups of Bees at Different Ages

In parallel to the aforementioned assay, a cage of each age group of bees (from 0 to 15 days p.e.) was used to inoculate bees collectively (Group B). To this end, a syrup solution was prepared containing purified spores (using the same final inoculum as above) at an equivalent dose of 5700 µL^−1^ spores in 400 µL of fresh syrup per 20 bee cage, assuming that each bee consumes 20 µL of food per day and that all bees consume the same amount. On the day of inoculation (spore administration), the feeders of all the Group B cages were replaced for 24 h with feeders containing the sucrose solution with the spores. Complete consumption of the food administered was verified after 24 h, such that the minimum age of the bees at the time of infection was considered as 1 day p.e. and thus, a cohort of 0 days was not available for the assay of Group B (Table 1). The food was then replaced with spore-free syrup (sucrose solution), which was administered *ad libitum* thereafter. In addition, control bees of 1, 7, 8, 10, 11 and 14 days p.e. that were not inoculated with spores (1 cage of 20 bees of each age received 400 µL of fresh syrup without spores) were also included to determine the absence of initial infection. Note that in these studies, no bees aged 4, 5 and 12 days p.e. were available. The cages with infected bees were kept in an incubator distinct to that used for Group A and again, physically separated to avoid cross-infection. The cages with uninfected bees were kept in a different incubator next to the cages with uninfected bees of Group A.

### 2.4. Molecular Detection of N. ceranae Infection (DNA Extraction and qPCR Analysis)

On day 7 post inoculation (p.i.), the number of surviving bees was recorded and they were frozen at −20 °C. When available, 10 bees were taken from each Group A (30 bees per cohort) and Group B (10 bees per cohort) cage, and each bee’s abdomen was carefully separated from the thorax and was placed individually into one well of a 96-well plate. Each of these wells contained four 2 mm glass beads (Sigma^®^) and 180 µL of sterile H_2_OmiliQ^®^ to homogenize the tissue by stirring at 30 Hz for 6 min (TyssueLyser II, Qiagen^®^). DNA was extracted from the resulting macerate and 50 μL was transferred on ice to a new multi-well plate (Eppendorf^®^, Hamburg, Germany) containing 50 μL of a Tris-HCl lysis solution (10 mM Tris-HCl [pH 8.0], 1 mM EDTA) and 15 μL of Proteinase K (Qiagen^®^, No. 1019499). The plates were incubated for 20 min at 95 °C in a Mastercycler ep Gradient S (Eppendorf^®^), with a well left with no sample between the bees from different cohorts and used as a DNA-extraction control (with H_2_OmiliQ^®^ and all the reagents: EC). *Nosema ceranae* was assessed by quantitative real time-PCR (qPCR) to detect DNA for the *polar tubule protein 3* (*PTP3*) gene [55] in a Roche LightCycler^®^ 480 thermocycler. The qPCR was performed in 384 plates and in a final reaction volume of 10 μL, using the LightCycler^®^ 480 Probes Master Mix (Roche) according to the manufacturer’s instructions: primers at 500 nM and universal probes at 10 nM (UPL, Roche Molecular Systems, Basel, Switzerland). Each amplification cycle was analyzed with the LightCycler^®^ 480 software v1.5.1 (Roche Diagnostics GmbH, Basel, Switzerland) and the crossing point (Cp) was recorded in all samples. Two replicates for each sample were included in the same kinetic qPCR run (intra-assay variation). The Cp value was calculated for each qPCR reaction from the standard curve using the second maximum derivative method.

The parasitic load was quantified in all samples relative to the specific synthetic oligonucleotides (gBlocks^®^, IDT DNA Technologies, Coralville, Iowa, USA) for the *N. ceranae PTP3* gene fragment (the sequence of the *PTP3* gBlock^®^ Gene Fragment is shown in Table 2), as designed using the gBlock^®^ Gene Fragment ordering tool. The non-variable areas of the gene were selected based on the sequence data available in GenBank and our in-house database [66]. The standard curve for quantification was prepared following the manufacturer’s protocol at an initial concentration of 10 ng/μL (in TE) and using serial dilutions up to 1 × 10^−14^ ng/µL.

To detect possible contamination in each qPCR reaction, the ECs and non-template controls (NTC) were analyzed in parallel in the same reaction plate. In addition, a positive control with DNA from *N. ceranae* extracted from naturally infected bees was included in each reaction to detect possible amplification failures.

### 2.5. Statistical Analysis: N. ceranae Infection According to Bee Age and the Mode of Infection

The average infection of *N. ceranae* was calculated in all the available bee cohorts and for each study group (Table 1) based on the average concentration value of the *N. ceranae-PTP3* gene at 7 days p.i. For each age cohort tested, 30 bee abdomens from Group A and 10 bee abdomens from Group B were analyzed individually.

To evaluate the possible effect of age and the method of infection on the extent of infection for each group of bees tested, generalized linear models (GLMs) were used. These GLMs allow variables to be modeled that do not meet the requirements for standard linear models (i.e., normality and homoscedasticity: Levene’s test), thus permitting the modeling of variables that follow distributions other than a normal distribution. In this study, the distribution that best fit the response variable-average DNA concentrations of *N. ceranae* was considered to be the gamma distribution (GLMz log link function).

To determine whether the “cage” (random factor) significantly affected the extent of infection within each age cohort (fixed factor) in Group A (3 cages per age cohort) a mixed GLM (GLMMz) was used. The infection levels were then compared in three different ways: (a) to evaluate the effect of the age cohort, the method of infection and their interaction in the study (Group A and Group B) by GLMz; (b) between different age cohorts within the same assay (Group A or Group B) using a Kruskal-Wallis and post-hoc Mann-Whitney-U test; and (c) between both assays (Group A vs. Group B) also using a Mann-Whitney-U test to compare the pooled data (n = 423 and n = 108 for Group A and B, respectively). *p* values <0.05 were considered significant and all statistical analyses were carried out using the IBM SPSS Statistics V24 software by the Statistics Unit of the Scientific Computing Area at the SGAI-CSIC (Madrid, Spain).

## 3. Results

All the bees used in the study were negative for *Nosema* spp. infection at birth, as were all the bees in the control groups (Group CA: n = 46; Group CB: n = 17) throughout. Likewise, no *Nosema* spp. were detected in the controls at the DNA extraction or PCR steps (EC and NTC), indicating that there was no cross-contamination during the molecular analysis.

The sensitivity of the qPCR was 1 × 10^−11^ ng/µL of *N. ceranae*-PTP3 DNA and the number of bees analyzed individually from each infection group (A and B) and from each age group is shown in Table 3.

Inoculation was more successful in the bees infected individually (n = 424; 99.76%) than when they were infected collectively (n = 130; 90%). In fact, virtually every bee exposed individually to the spores was infected (Group A), irrespective of their age, whereas there was considerably more variation in the rates of infection found in Group B, with the lowest levels of infection detected in bees exposed to the spores at 13 days p.e. and especially at 15 days p.e., when only one bee was infected 7 days p.i. (Table 3). By contrast, all bees were infected when they were inoculated at 16 days p.e.

### 3.1. Level of Infection in Bees of Different Ages (Group A)

**Figure 1:**
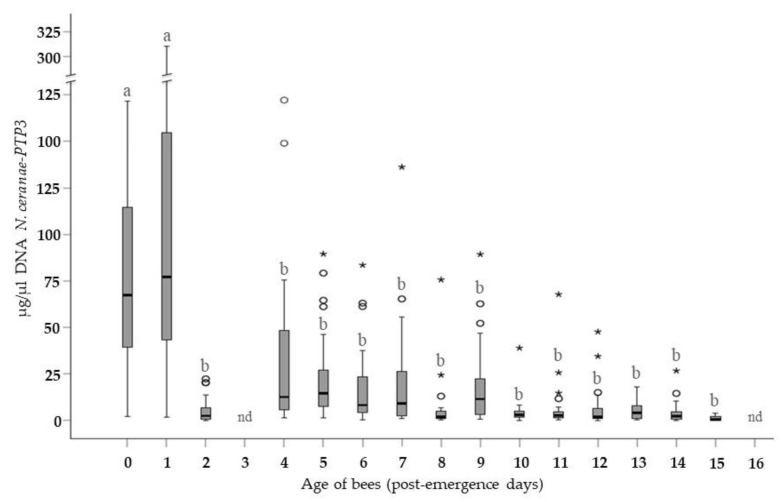
Average concentration of the *N. ceranae*-PTP3 DNA (µg/µL) in Group A bees 7 days p.i. The letters (a, b) indicate statistical significance *p* < 0.05 (Mann-Whitney-U test). *: extreme cases (any value > Q1 − 1.5 * IQR or > Q3 + 1.5 * IQR; Q: quartile; IQR: interquartile range). o: very unlikely cases (any value > Q1 – 3 * IQR or > Q3 + 3 * IQR; Q: quartile; IQR: interquartile range). Extreme cases and very unlikely cases were calculated separately within each age. nd: no data.

The average level of infection of Group A bees was analyzed, and as can be seen (Figure 1), the age of the bees at the time of exposure to the spores significantly influenced the degree of infection evident at 7 days p.i. (GLMz, Wald Chi-Square test; *p* < 0.001). The largest amount of *N. ceranae*-PTP3 DNA amplified was obtained from bees infected at ages 0–24 h p.e. Infection was a significantly milder in bees exposed to the spores at 48 h p.e. (Mann-Whitney test, *p* ≤ 0.05), leading to an approximately 20-fold reduction in the parasite load than in the younger bees. The level of infection again increased in bees exposed at 4 days p.e., although this increase was not associated with a significant rise in the parasitic load relative to the bees exposed to the spores on day 2 p.e. However, the levels were lower at all the remaining ages. The parasitic load remained low in the cohorts exposed between 4 and 9 days p.e., with the lowest levels detected in infected bees exposed to the spores at more advanced ages, from 10 to 15 days p.e. Thus, there was an indirect trend towards lower levels of infection as the age of the bees increased, which was up to an order of magnitude lower than that of the immediately younger bees. The average concentration of the pathogen (µg/µL) was statistically different between the bees at 0 and 1 days p.e. when compared to the rest of the cohort.

The mean standard deviation observed in the cohorts studied was relatively high due to the large variation in the degree of parasitization between each individual bee within the same age group. The effect of the “cage” on the response was apparently not significant (GLMMz, Wald Chi-Squared test: *p* = 0.582) and thus, the average DNA concentration of *N. ceranae* was not modified when the 30 values registered from bees of the same age were grouped.

### 3.2. Level of Infection According to Age and Method of Infection (Group A vs. Group B)

**Figure 2:**
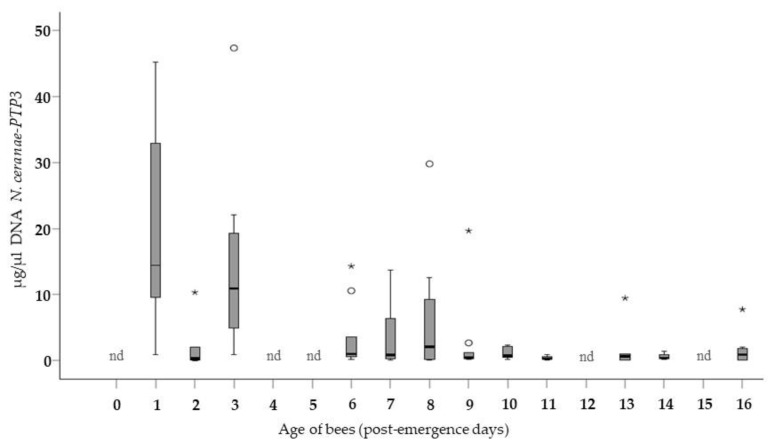
Average concentration of the *N. ceranae*-PTP3 DNA (µg/µL) 7 days after the collective infection of the bees in Group B: *: extreme cases (any value > Q1 − 1.5 * IQR or > Q3 + 1.5 * IQR; Q: quartile; IQR: interquartile range). o: very unlikely cases (any value > Q1 − 3 * IQR or > Q3 + 3 * IQR; Q: quartile; IQR: interquartile range). Extreme cases and very unlikely cases were calculated separately within each age. nd: no data. As indicated, bees were exposed collectively to the *N. ceranae* spores on day 0 p.e. and analyzed 24 h later (after consuming the contaminated food).

When the average concentration of the *N. ceranae*-PTP3 gene DNA was assessed in all the collectively inoculated cohorts (Figure 2), the data confirmed the trend observed in the bees infected individually (Figure 1). Thus, the evolution of the infection in the Group B bees was very similar to that in Group A, and the interaction effect (age * method) was not significant (GLMz, Wald Chi-Squared; *p* = 0.75). Accordingly, when the youngest bees were infected (1 day p.e.) they had more *N. ceranae* DNA than those bees that were exposed to the spores later in life, at 2 days p.e. and older. Bees exposed to the parasite at 2 days p.e. developed microsporidium infection on average ≈8 times less than that of the bees exposed to the spores on day 1 p.e. The average parasitization of the bees infected at 3 days p.e. was greater than in those infected on the previous day, yet not higher than that registered in bees exposed to the parasite on day 1 p.e. Moreover, this level was maintained in the infected cohorts until 10 days p.e. The lowest average levels of infection was detected in the more mature bees, those exposed to the parasite from 11 to 16 days p.e. In bees of these ages, the values were an order of magnitude lower than in the immediately younger bees, confirming that less *N. ceranae* DNA was detected as the age at infection increased. In this group of collectively infected bees, the distribution of the average concentration was not statistically different among the bees inoculated at the different ages.

The levels of infection in Group A (Figure 1) were generally higher than those in Group B (Figure 2) when bees exposed to the spores at the same age were compared. When the values were grouped according to the method of infection (Group A vs. Group B), a significantly higher average concentration of microsporidia (Mann-Whitney-U Test: *p* = 0.004) was observed in the individually infected bees relative to those infected collectively. The largest amount of *N. ceranae* DNA in the entire study (7 days p.i.) was observed in the bees infected individually at the age of 1 day p.e.

## 4. Discussion

The objective of this study was to assess how the age at the time of inoculation, and the method used for this influences the parasite infection that develops. The *N. ceranae* parasitic load was quantified without analyzing its effect on the viability of the bees. Accordingly, it was evident that both these factors, the age of the bee and the method of inoculation, affect the development of *N. ceranae* infection.

In this study, a dose of spores was administered that was expected to ensure 100% infection of the bees [25,44,61,64,67,68] using spores from different naturally infected colonies to favor the natural heterogeneity of the regional population of *N. ceranae* [24,69,70]. The study design involved assessing infection at 7 days p.i. to prevent the proliferation of spores to equal levels between all groups over longer periods [71], and to assess the infection at a specific time point before many of the bees had died due to the infection [18,72]. Thus, we avoided the prolonged confinement that increases both the levels of *Nosema spp.* infection [73] and bee mortality [18,72].

The results obtained here demonstrate that the success of infection under controlled conditions and/or the ability of *N. ceranae* to multiply depend significantly on the age of the bees at the time of infection, as well as on the method of spore administration. The youngest adult bees were especially susceptible to infection (0–1 day p.e.) and the parasitic load at 7 days p.i. was significantly higher following exposure to spores at that age. A lower parasitic *N. ceranae* load was detected in bees infected at 2 days p.e., a trend that was observed in both study groups (individually and collectively infected). The differences in the level of infection of the bees of different ages cannot be attributed to the origin of the spores or bees, since all were fed with the same *N. ceranae* spore mixture and all came from the same breeding frames.

In agreement with these results, Chaimanee et al. [48] showed that queen bees infected collectively 1 day p.e. were more susceptible to infection by *N. ceranae* and they developed significantly higher levels of infection than queen bees infected at 6 and 12 days p.e. Conversely, Huang et al. [25] reported that newly emerged bees were the least susceptible to *N. ceranae* whereas bees infected at 5 days p.e. were more susceptible to these parasites, and older honey bees infected by *N. ceranae* developed more intense infections (spore number at 12 days p.i.) and survived better than younger bees [74].

It is known that bees undergo particularly important natural modifications in the composition and succession of their intestinal microbiota over the initial days of their adult development. Indeed, new-born bees begin to acquire their intestinal microbiota within a matter of hours and they are fully colonized by the sixth day p.e. [75,76,77,78]. The bacterial community that colonizes the bee gut is resilient to changes in the nutritional, hive, and social environment [77]. However, it has been suggested that active microbiota differ in species richness and total abundance across the ontogenetic stage of honey bee and hive location [78], as has also been proposed for many other animal species. Consequently, the success of *N. ceranae* infection may be related to the microbiota becoming established in the intestine of the bees and its competition with the parasite for the host’s resources, as well as to the physiological events inherent to bee development [49,50]. These modifications could affect the biological cycle of microsporidia [79] and perhaps the intensity of infection [80]. In fact, the promotion of diseases in honey bees, and therefore the health of the colony, may be associated with changes in the composition and abundance of the intestinal microbiota of individual bees [79,81,82,83,84]. This phenomenon might explain why the greatest differences in infection were seen here among the youngest cohorts of 0 and 1 days p.e. However, the relative abundance of some members of the microbiota is associated with the environment in which they develop [85], and the succession of communities is strongly associated with the breed and age of the bees [86]. In our study, the bees were kept in an incubator and not in the colony, which most likely influences the establishment of normal microbiota. Moreover, the strict dependence on host energy for the development of microsporidia [87,88] and the greater availability of essential amino acids in young bees [89] or even the absence of a well-established peritrophic membrane could also favor the multiplication of the parasite in younger bees [87,88,90].

Other elements that are likely to influence the pathogenic events mediated by *N. ceranae* infection include the host’s immune response, which varies with age through a phenomenon generally referred to as immunosenescence (reviewed in [90]). This senescence often weakens the immune response, either cellular (capsulation, nodulation or melanization) or humoral (antimicrobial peptide synthesis and oxidative response), frequently enhancing the intensity of infection in older insects [91]. Indeed, older honey bees develop a more intense *N. ceranae* infection than younger bees, producing less prophenoloxidase, which is also negatively correlated with the intensity of parasite infection [74]. However, it is not yet clear what effect the innate humoral response of honey bees has on the extent of *N. ceranae* infection, particularly how the microsporidia are affected by antimicrobial peptides and chemical signaling cascades. This may be because the parasite passes directly through the intestinal lumen to the midgut epithelial layer where the infection develops, and the effects of immune peptides and chemical cascades are less likely to reach the parasite at this location. In fact, one host cell mechanism used to combat intracellular parasites is the apoptosis of infected cells, which is dampened following infection by *N. ceranae* and other microsporidia [92,93,94].

It is commonly accepted that the older forager bees in hives have the highest frequency and most intense infection [45,46,95,96], which is thought to provoke an acceleration of foraging behavior in bees infected by *N. ceranae* [32,33,34,58,97]. However, given the greater susceptibility of younger worker bees evident in this study, it is possible that various strategies may be employed in the super-organism to reduce contamination by *Nosema* spp. in the nucleus of the hive where they develop. Social insects are known to develop social immunity, which is expressed through a variety of sanitary behaviors and the use of antimicrobials to reduce the risk of infection and the pathogen load of exposed individuals [98]. Some interactions among colony members also contribute to limiting pathogen spread at the colony-level and to decrease the infection risk of valuable individuals, providing “organizational immunity” [98]. In fact, bee colonies are highly compartmentalized structures, and the connectivity and spatial overlap is considerable among same-age worker bees, yet weak among different-age workers [99]. In the case of *N. ceranae*, infection increased the frequency of antennal contact [100], probably because infected bees solicit more food as they are more responsive to sucrose and less inclined to share this food with other bees, a response that might influence the transmission of infection [101]. On the other hand, the spatial distribution of various age classes established in the bee colony reflects a degree of immunity in young individuals [102]. Foragers (the most intensely infected population in a colony) are at the periphery of the social network, while the colony core is formed by young bees and the queen [99]. As such, the latter are protected from potentially harmful external agents [98], such as *N. ceranae* spores. As they age, worker bees shift from tasks in the interior to peripheral tasks, like food processing and nest maintenance, and at the end of their lives they perform outside-nest tasks [98]. However, *N. ceranae* infection produces behavioral and physiological changes in bees [100,103], inducing infected bees to become early foragers and thereby delaying the onset of foraging in their healthy nest-mates [103]. This has been proposed to as defense mechanism in infected colonies, decreasing the spatial overlap of healthy bees with infected workers and delaying the drain on the nursing force induced by *Nosema* infection [98]. This whole battery of strategies has been proposed to prevent the infection of young queen bees [48], young worker bees [104] and immature bees [68,105], which have a lower risk of developing the disease even when the prevalence of infection in adult bees is relatively high [55].

In addition to the biological factors associated with the different life stages of the bees, *N. ceranae* infection itself can affect different aspects of the physiology (metabolism and immune response), morphology and behavior of honey bees [21,34,92,105,106,107], thereby influencing the viability and intensity of the infection. Some evidence suggests that parasite manipulation of intestinal homeostasis and the inhibition of intestinal epithelial renewal [92,108] are correlated with an increased susceptibility of bees to *N. ceranae* infection due to their diminished capacity to repair the intestinal damage [108]. Ventricular epithelial cells in young bees 1–2 days p.e. are still undergoing morphogenetic development [109], which may be associated with the ability to accumulate infected apoptotic cells in the midgut and rectum when they are experimentally infected [94].

Although the collective assay (Group B) was carried out on just one cage while three cages were used for the individual assay (Group A), a similar trend was observed in both assays. The prevalence of infection and the average levels of infection were significantly higher in individually infected bees than in those infected collectively, which may explain some of the conflicting results found in the literature. In this sense, important differences in food intake have been described between bees caged together within the first 48 h [110]. These observations suggest bees are exposed to distinct numbers of *N. ceranae* spores during collective infection. Indeed, in some studies only 23.3% [111] or 78% [32] of caged bees become infected 12–15 days after collective administration of spores in food.

Trophallaxis favors the transmission of spores among bees and it has been shown that while only three infected bees were found in five samples analyzed in the inoculated cages at 5 days p.i., all bees sampled at 10 and 15 days p.i. were infected [112]. However, infected bees may be less willing to share food with their cage mates [101] or conversely, their trophallactic behavior may be enhanced [32]. In addition, the number of infected bees in a cage and their parasite load are factors that influence the exchange of food between infected and uninfected bees [113].

The levels of *N. ceranae* infection in both groups (Group A and Group B) showed similar trends, with significantly higher values in bees exposed to spores in the first 48 h p.e. and lower values as the bees aged. Therefore, considering all the variables that could modulate the frequency and intensity of infection, and the exchange of contaminated food between caged bees, we consider that the age of the bee at the time of infection influences the degree of infection and the development of nosemosis, as suggested for *N. apis* infection [104]. In addition, the number of uninfected bees following collective exposure to the spores was higher than that of individual infections, particularly when old bees (11–15 days p.e.) were infected. These phenomena could affect the interpretation of the results of experimental studies, and thus, both these variables must be considered in laboratory studies. Likewise, in addition to cage experiments, more field tests should be carried out as differences have been found in the morphology, physiology, behavior and gene expression of honey bees (workers and queens) reared in the laboratory when compared to those naturally infected by microsporidia [81,114,115,116,117].

## 5. Conclusions

The bees infected in the first 48 h p.e. develop significantly high levels of *N. ceranae* infection and as their age increases, there is a trend towards milder infection independent of the method used to administer the spores. Hence, younger bees appear to be more susceptible to infection and/or the microsporidia is more capable of multiplying better in these bees. Moreover, our results demonstrate that the effect of age on *N. ceranae* infection is similar in bees exposed individual or collectively. Collective inoculation was less successful and the bees, when infected, had lower parasitic charge. Thus, the results presented here demonstrate that bee age and the method of inoculation ultimately determine the extent of infection, which is important to take into account when comparing experimental studies carried out in the laboratory. Future research aimed at elucidating the physiological differences between bees of different ages might help explain their different susceptibility to *N. ceranae* infection, potential paving the way to design strategies to combat this pathogen.

## Figures and Tables

**Table 1:** Total number of bees in each cohort and infection type.

	Experimental Design																
**Age Cohort**	**0**	**1**	**2**	**3**	**4**	**5**	**6**	**7**	**8**	**9**	**10**	**11**	**12**	**13**	**14**	**15**	**16**
**Group A**	90	60	60	-	60	60	60	60	60	60	60	60	60	60	60	60	-
**Group CA**	20	20	-	-	20	20	-	-	20	-	-	20	-	20	20	-	-
**Group B**	-	20	20	20	-	-	20	20	20	20	20	20	-	20	20	20	20
**Group CB**	-	20	-	-	-	-	-	20	20	-	20	20	-	-	20	-	-

Group A, individually infected bees (n = 20 bees × 3 cages), Group B, collectively infected bees (n = 20 bees × 1 cage) and uninfected control Groups: CA (individually fed with spore-free water) and CB (collectively fed with the plain sucrose solution). Hyphen (-) indicates bees not available. The bees that died were removed from the study.

**Table 2:** *PTP3* gBlock^®^ Gene Fragment.

Method	Organism	*PTP3 gBlock^®^ Gene Fragment*	Source
qPCR	*N. ceranae*	5′_TGAAGCTAAAAAAGAAGAACAACTTGACCAAATAGCTAAAAAGAATGCAGAGACAGAGAAACAACACAGAGAGGTACTTCTCAAAGAACATCAAGA**TGCTGATGTTATGGCTACAGAAGA**AAGACTTGCTAAAAATAATAGAgccaggaaGATTAGTGAGGCAGGAA**TTAAAGCAGCGCAATCTGTA**TTGAAAACTGGAGGAACAATAGAAGAAGCAAGAGCAGCTAAGGCGGCAGCTGAAAAAGCTATATTGCAAGAAATTGAGAGTAGAGAAGCGCAAA_3′	gi|557790804|gb|KC520145.1|

A 281 nucleotide fragment of the *N. ceranae* genome including the NC-PTP3 primer sequences (underlined in bold), and the sequence of the UPL#72 probe in bold and lowercase.

**Table 3:** Number of bees and the percentage of bees infected by *N. ceranae* at 7 days post-inoculation (p.i.).

		Infected Bees (7 Days p.i.)																
**Age Cohort**		**0**	**1**	**2**	**3**	**4**	**5**	**6**	**7**	**8**	**9**	**10**	**11**	**12**	**13**	**14**	**15**	**16**
**Group A**	n	30	30	24	-	30	30	30	30	21	30	30	30	30	30	30	19 *	-
	qPCR+	30	29	24	-	30	30	30	30	21	30	30	30	30	30	30	19	-
	**%**	**100**	**97**	**100**	**-**	**100**	**100**	**100**	**100**	**100**	**100**	**100**	**100**	**100**	**100**	**100**	**100**	**-**
**Group B**	n	-	10	10	10	-	-	10	10	10	10	10	10	-	10	10	10	10
	qPCR+	-	10	9	10	-	-	10	10	9	10	9	8	-	5	8	1	10
	**%**	**-**	**100**	**90**	**100**	**-**	**-**	**100**	**100**	**90**	**100**	**90**	**80**	**-**	**50**	**80**	**10**	**100**

Group A, bees infected individually with *N. ceranae* spores; (*) n < 30 bees due to the high accumulated mortality of the bees at 7 days p.i. Group B, bees infected collectively with the same spores. All control bees analyzed (Group CA and Group CB) were negative for *N. ceranae* infection and they are not shown in the table. n: number of samples analyzed. qPCR +: indicates number of positive samples.
